# An Updated Systematic Literature Review of Lay Support Models in the Management of Postnatal Depression: Evidence from 2010 to 2024

**DOI:** 10.1192/j.eurpsy.2026.12047

**Published:** 2026-07-31

**Authors:** L. Colbourne

**Affiliations:** Liaison Psychiatry, Kingston Hospital, South West London & St George’s Mental Health Trust, London, United Kingdom

## Abstract

**Introduction:**

In recent years, there has been increasing attention on the potential of lay health workers and peer support models to address PND. These interventions are considered cost-effective, culturally appropriate, and accessible alternatives to traditional mental health services. This review serves as an update to a previous literature review (Potter CL. *European Psychiatry.* 2011;26(S2):1105-1105) that covered studies published between 1980 and 2010, focusing on the new evidence that has emerged since then.

**Objectives:**

This review aimed to evaluate published evidence on the effectiveness of lay support models for managing postnatal depression (PND), with a focus on studies published between 2010 and 2024. It was hypothesised that lay support interventions may benefit maternal mental health, with effectiveness varying by intervention type and target population.

**Methods:**

A systematic review was conducted using MEDLINE, EMBASE, PsycINFO, and the Cochrane Library to identify randomised controlled trials (RCTs) published from August 2010 to October 2024. Studies included in this review focused on lay-led interventions for postnatal women, with outcomes related to PND, maternal mental health, and self-esteem. A total of 42 studies were included in the review.

**Results:**

The findings highlight the varied effectiveness of lay support models. Peer-led telephone support, especially for high-risk groups, showed significant reductions in depressive symptoms, as measured by the Edinburgh Postnatal Depression Scale (EPDS). Similarly, mindfulness-based childbirth courses and doula services demonstrated improvements in maternal self-efficacy and infant care practices, although they did not consistently reduce depressive symptoms. Interventions in low- and middle-income countries, such as group interpersonal therapy (g-IPT) and peer-led women’s groups, were associated with improved maternal mental health and stronger mother-infant bonding. Digital support models, including telemedicine and peer counselling, proved effective in settings with limited face-to-face care options. However, some interventions, including self-help manuals and community group support, showed no significant impact on PND.

**Conclusions:**

This review provides evidence that lay support models can offer benefits for managing PND, particularly for high-risk and underserved populations. However, the effectiveness of different interventions varies, and further research is needed to determine which approaches are most effective in diverse cultural and socioeconomic contexts. The findings suggest that while lay support can be a promising strategy, additional studies are required to better understand its long-term impacts and cost-effectiveness.

**Disclosure of Interest:**

None Declared

